# Humans and climate change drove the Holocene decline of the brown bear

**DOI:** 10.1038/s41598-017-10772-6

**Published:** 2017-09-04

**Authors:** Jörg Albrecht, Kamil A. Bartoń, Nuria Selva, Robert S. Sommer, Jon E. Swenson, Richard Bischof

**Affiliations:** 1 Institute of Nature Conservation, Polish Academy of Sciences, Mickiewicza 33, PL-31-120 Kraków, Poland; 2 Department of Zoology, Institute of Bioscience, University of Rostock, Universitätsplatz 2, D-18055 Rostock, Germany; 3 Faculty of Environmental Sciences and Natural Resource Management, Norwegian University of Life Sciences, PO Box 5003, NO-1432 Ås, Norway; 4 Norwegian Institute for Nature Research, NO-7485 Trondheim, Norway; 5 Senckenberg Biodiversity and Climate Research Centre (BiK-F), Senckenberganlage 25, 60325 Frankfurt am Main, Germany

## Abstract

The current debate about megafaunal extinctions during the Quaternary focuses on the extent to which they were driven by humans, climate change, or both. These two factors may have interacted in a complex and unexpected manner, leaving the exact pathways to prehistoric extinctions unresolved. Here we quantify, with unprecedented detail, the contribution of humans and climate change to the Holocene decline of the largest living terrestrial carnivore, the brown bear (*Ursus arctos*), on a continental scale. We inform a spatially explicit metapopulation model for the species by combining life-history data and an extensive archaeofaunal record from excavations across Europe with reconstructed climate and land-use data reaching back 12,000 years. The model reveals that, despite the broad climatic niche of the brown bear, increasing winter temperatures contributed substantially to its Holocene decline *—* both directly by reducing the species’ reproductive rate and indirectly by facilitating human land use. The first local extinctions occurred during the Mid-Holocene warming period, but the rise of the Roman Empire 2,000 years ago marked the onset of large-scale extinctions, followed by increasingly rapid range loss and fragmentation. These findings strongly support the hypothesis that complex interactions between climate and humans may have accelerated megafaunal extinctions.

## Introduction

Megafaunal extinctions are a hallmark of the late Pleistocene and early Holocene, with evidence suggesting human expansion and climatic changes as the main causes^1–9^. Yet, the direct and indirect contributions of these two drivers to prehistoric species extinctions generally remain unclear when human expansions co-occurred with major climatic shifts^10–12^. On one hand, periods of warming climate could have facilitated the colonization of previously inhospitable regions by humans^13–15^, with species extinctions following human arrival. On the other hand, climate change may have promoted species extinctions through a decrease in resource availability^11^ or through direct effects on key life-history traits^16, 17^. Disentangling these complex relationships requires approaches that integrate the spatial and temporal dimensions of species extinctions^18^.

Here we resolve the contribution of humans and climate change to the population decline of the largest living terrestrial carnivore, the brown bear (*Ursus arctos*), on the European continent during the Holocene. While present throughout the continent during the Late Glacial^19^, today the species is confined to a few isolated relic populations^20^. Human land use and persecution are known to have played a key role in the range contraction of this large carnivore^21, 22^, but the contribution of climate warming during the Holocene is unknown.

As a hibernator, the brown bear is adapted to seasonal climates with prolonged periods of energetic bottlenecks^16^ (Supplementary Fig. 1). Because female brown bears give birth during the hibernation period^23^, they face a trade-off between overwinter survival and reproduction. The allocation of energy to either depends primarily on the energy reserves at the onset of hibernation^23^ and on the energy required to survive the winter. Paradoxically, bioenergetic models predict that energy demands of hibernating mammals increase during warm winters, because the energetic costs of torpor increase^16^. This means that less energy can be allocated to reproduction during warm winters (Supplementary Fig. 1). To compensate for this loss of reproductive potential, brown bears must increase energy uptake during the growing season, when they feed extensively on plant material (e.g., berries and seeds) to build up body fat before den entry^23^. Therefore, net primary productivity and winter temperature should impose a strong life-history trade-off for the reproductive rate of the brown bear, which could have directly affected the population dynamics of the species during the warming period after the Late Glacial.

## Results and Discussion

**Figure 1:**
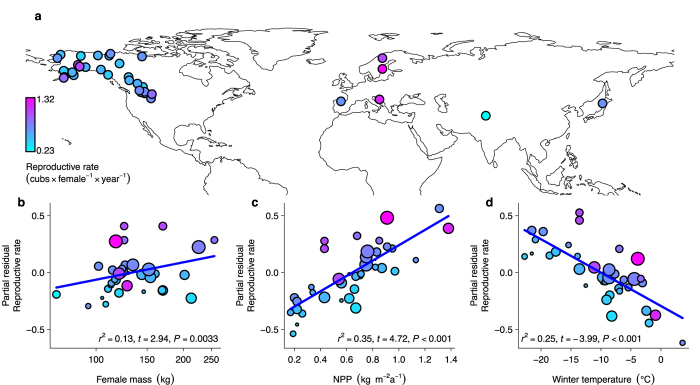
Relationships of body mass, net primary productivity and winter temperature with the reproductive rate of female brown bears. (**a**) Map showing the locations of 38 brown bear populations included in the analysis with their reproductive rate estimated as cubs × female^–1^ × year^–1^. (**b**–**d**), Partial relationships of reproductive rate with (**b**) mean female body mass, (**c**) net primary productivity, and (**c**) mean winter temperature after accounting for the other respective variables. In (**b**–**d**), partial *r* ^2^–values indicate independent contributions of the respective variables. The size of the circles in (**b**–**d**) is proportional to the weight of the observations in the analysis (see Methods section). Circle colour represents the mean reproductive rate ranging from low (cyan) to high (magenta). The map in (**a**) was created with the statistical programming language *R* ^68^ using the package *rworldmap* ^72^.

To test this hypothesis, we first compiled data about the life histories of female brown bears from 38 populations across the species’ entire geographic range (Fig. 1a). We found that reproductive rate increases with net primary productivity, but decreases with increasing winter temperature (Fig. 1b,c; Supplementary Table 1). This suggests that, due to the trade-off between overwinter survival and reproduction, changes in net primary productivity and winter temperature during the Holocene directly affected the population dynamics of the brown bear by reducing the species’ reproductive rate.

**Figure 2:**
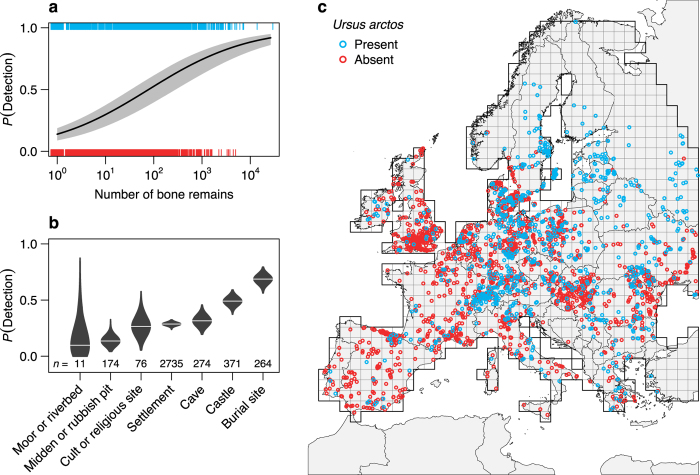
Occurrence of brown bear bone remains and their detection probability in archaeofaunal records across Europe. (**a**,**b**), Brown bear detection probability estimated by the metapopulation model (**a**) as a function of the total number of bone remains of red deer (*Cervus elaphus*), roe deer (*Capreolus capreolus*) and brown bear per record, and (**b**) as a function of the type of excavation site. The model estimated detection probability based on whether or not brown bear bone remains had been detected in (**c**) archaeofaunal records on a 100 × 100 km^2^ grid over Europe during the past 12,000 years. Records containing red deer or roe deer, but not brown bear, served as negative controls, because both deer species were typical elements of the European fauna during the Holocene and the most frequent prey of European Stone Age hunters^25, 26^. This approach allowed us to account for imperfect detection in the archaeofaunal record, while simultaneously modelling the spatiotemporal variation in brown bear extinction and colonization rates across the European continent. Brown bear bone remains were present in 27% of the archaeofaunal records. The probability of detection increased with the total number of bone remains contained in a record (**a**), and was highest in records associated with burial sites (**b**), which reflects rituals and beliefs associated with bears in human cultures in the northern hemisphere^55^. (**c**) The distribution of the archaeofaunal records (*n* = 4,177) and the grid (cell size: 100 × 100 km^2^) that was used for the metapopulation model. Ticks in (**a**), and circles in (**c**) represent individual archaeofaunal records with (blue) and without (red) remains of brown bears. In (**a**), the black line represents the median model fit; the gray shade depicts the 95% credible interval from the posterior distribution. In (**b**), the violins represent the density distributions of estimated detection probability for each site type. The map in (**c**) was created with the statistical programming language *R* ^68^ using the package *rworldxtra* ^73^.

Next, we aimed to decompose the effects of Holocene changes in net primary productivity, winter temperature, and human land use (a proxy for habitat loss and persecution)^11^ on the population dynamics of the brown bear on the European continent. To do so, we developed a Bayesian hierarchical spatially explicit metapopulation model based on detection/non-detection data^24^. We informed the metapopulation model with 4,177 archaeofaunal records of subfossil bone remains from 3,461 excavation sites across Europe^19, 25–27^, extending back to the Late Glacial, approximately 12,000 years ago (Fig. 2; Supplementary Fig. 2). We complemented these records with data about the species’ distribution in the 1950–70 s, and at present^20^. The model accounted for imperfect detection due to differences in bone preservation^28^ and collection effort between excavation sites^4^ (Fig. 2a), as well as for heterogeneity in detection probabilities among different types of excavation sites (Fig. 2b).

**Figure 3:**
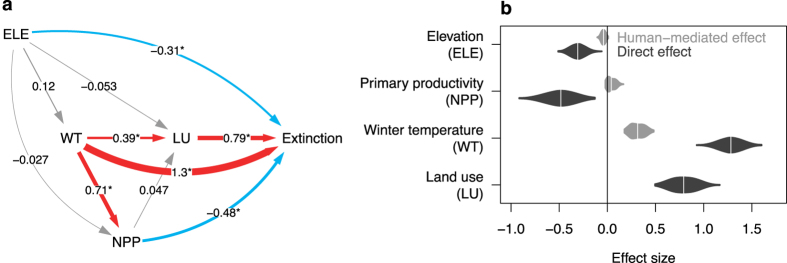
Direct and indirect effects of environmental conditions and human land use on the extinction rate of the brown bear in Europe during the past 12,000 years. (**a**) Path diagram and (**b**), direct effects of land use (LU), winter temperature (WT), net primary productivity (NPP), and elevation (ELE) on the extinction rate of the brown bear, as well as indirect effects of the three environmental variables mediated by humans. In (**a**), paths that emerged from Bayesian variable selection with a probability >95% are highlighted with an asterisk (Supplementary Table 2). Path widths are proportional to standardized effect sizes. In (**b**), the violins represent the density distributions of estimated direct and indirect effects for each explanatory variable. Note that positive effects (red paths) increase extinction rate, whereas negative effects (blue paths) decrease extinction rate. Uncertainty in model assumptions was incorporated by a two-factorial design with two scenarios for changes in per capita land-use intensity during the Holocene (constant *versus* decreasing) and two scenarios for colonization of the European continent by the brown bear from Asia (yes *versus* no), respectively (see Supplementary Methods).

We combined the metapopulation model with a path analysis to disentangle the potential direct and indirect causal effects of winter temperature, net primary productivity and human land use on the extinction rate of the brown bear (Fig. 3a). We further included elevation into the path analysis to assure that the effects of the other three variables were not simply an artefact caused by the role of montane habitats as refugia^29^. We used a Bayesian variable selection procedure to select the pathways that best explained the relationships among variables. Of 1,024 possible models (all combinations of 10 paths), 16 were selected at least once during the Markov Chain Monte Carlo search (Supplementary Table 2). All of the selected models indicated that increasing land use and winter temperature were the principal drivers of extinction, with humans partially mediating the effect of winter temperature (Fig. 3). This suggests that increasing winter temperatures facilitated human land use in regions with formerly unsuitable climates^13, 14^, thereby expediting overhunting and habitat loss^10^.

Most strikingly, the increase in winter temperature also increased the extinction rate directly (Fig. 3), which is in line with the predicted negative relationship between winter temperature and the reproductive rate of the brown bear (Fig. 1c). As expected, net primary productivity partly dampened the effect of winter temperature on the extinction rate by 26.5% (14.9–38.1%, 95% credible interval, CI hereafter). Increasing net primary productivity associated with rising temperatures after the Late Glacial may, thus, have facilitated the persistence and expansion of the brown bear in some regions. Yet, warming in Europe during the Mid-Holocene was greater in winter than in summer, with a 2 to 4 °C increase in most parts of Europe over the last 12,000 years^30^. This discrepancy between summer and winter warming might have limited the adaptation of the species to suboptimal wintering conditions. After accounting for the dampening effect of net primary productivity, the remaining direct effect of winter temperature (0.94, 0.77–1.1; mean, 95% CI) was of the same magnitude as the direct effect of human land use (0.79, 0.60–0.99; mean, 95% CI). Importantly, these estimates incorporate uncertainty in model assumptions about changes in per capita land use through time and about colonization of the European continent by the brown bear from Asia, respectively (see Supplementary Methods). Thus, even after accounting for these potential biases, our results still suggest that increasing winter temperatures contributed substantially to the Holocene decline of the brown bear in Europe.

**Figure 4:**
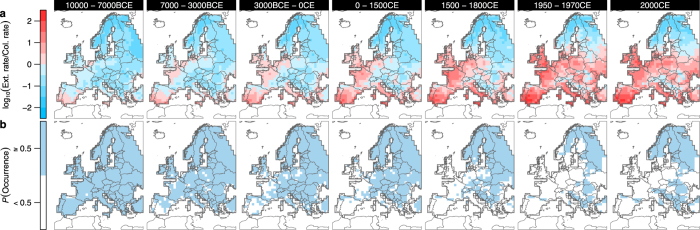
Spatiotemporal trends of extinction and colonization rates and occurrence of the brown bear in Europe during the past 12,000 years. (**a**,**b**), Maps showing (**a**) the net trend towards extinction (red) or colonization (blue) given as the logarithm of the ratio of extinction to colonization rates [log_10_(Ext. rate/Col. rate)], and (**b**) the probability of brown bear occurrence (blue) in each period. Estimates of extinction and colonization rates and occurrence probability were obtained from a metapopulation model based on archaeofaunal and contemporary occurrence records for the brown bear. Uncertainty in model assumptions was incorporated by a two-factorial design with two scenarios for changes in per capita land-use intensity during the Holocene (constant *versus* decreasing) and two scenarios for colonization of the European continent by the brown bear from Asia (yes *versus* no), respectively (see Supplementary Methods). The maps in (**a**,**b**) were created with the statistical programming language *R* ^68^ using the package *rworldxtra* ^73^.

The model predicts that the first local extinctions occurred in southwestern Europe during the Mid-Holocene warming period (ca. 7,000 to 5,000 years ago; Fig. 4). The rise of the Roman Empire, approximately 2,000 years ago, marked the onset of large-scale extinctions in southwestern Europe and on the British Isles, followed by further range loss in central Europe that ultimately led to today’s fragmented relic populations (Fig. 4). The spatiotemporal coincidence of increased extinction rate with the rise of the Roman Empire could stem from direct hunting or persecution to protect livestock^31^, from capture and killing for public entertainment^32^, or from large-scale habitat loss due to deforestation^33^. However, the spatiotemporal pattern of range contraction also reveals that local extinctions not only followed the increase in human land use, but also closely tracked the Holocene increase in winter temperatures across Europe (Fig. 4; Supplementary Fig. 3). Explicitly considering the spatial information in the distribution of archaeofaunal and contemporary occurrence records allowed us to disentangle the direct and indirect effects of environmental factors on the decline of the brown bear. Therefore, and importantly, direct effects of climate on megafaunal extinctions may not be evident where information on the spatiotemporal dynamics of species occurrences is not available^10^.

Our study yields perhaps the most comprehensive glimpse into the spatial and temporal patterns of a species’ extinction in the distant past. We demonstrate that considering spatiotemporal dynamics explicitly can provide unique and more detailed insights into the causes of prehistoric extinctions than purely chronological data. We conclude that, apart from human impacts, the direct interaction of changing climatic conditions with species’ life histories is a critical pathway to species extinctions, both in the past and today. Therefore, our analysis strongly suggests that, when climatic shifts coincided with human expansions, the mere dichotomy of ‘humans *versus* climate’ may often be insufficient to explain megafaunal extinctions^10, 11^. More plausibly, complex direct and indirect effects of humans and changing environmental conditions caused the prehistoric megafaunal extinctions that demarcate the onset of the defaunation of Earth’s ecosystems.

## Methods

### Meta-analysis

Reproduction imposes one of the most expensive energetic costs on organisms with parental care, such as mammals^34^. Life-history theory predicts that resource availability and metabolic demands cause a trade-off between life-history traits related to adult survival and reproduction. Low resource availability or high metabolic demands may both force an organism to invest in self-survival, but at the cost of reproduction^34^. Because life-history strategies are typically under strong environmental selection^34^, comparative approaches that investigate how investment in reproduction varies across populations in contrasting environments can offer insights into the factors that cause life-history trade-offs.

We used a meta-analysis to test the hypothesis that energy availability during the growing season and energy expenditure during the wintering season impose a life-history trade-off on the reproductive rate of the brown bear (*Ursus arctos*). To do so, we compiled information on litter size (*LS*, mean number of cubs per litter), inter-birth interval (*LI*, mean number of years between litters), and female body mass (*FM*, kg) for 43 brown bear populations from North America (*n* = 33), Europe (*n* = 6) and Asia (*n* = 4) from 25 published studies (Supplementary Table 3). We excluded five populations where data was not available for all variables from the analysis. Based on the reported litter sizes and inter-birth intervals, we calculated the reproductive rate as *RR* = *LS*/*LI* (cubs per female per year) for each of the remaining 38 populations.

We compiled the minimum, maximum, and average monthly temperatures and average monthly precipitation at the locations of the study populations (spatial resolution of 2° × 2°; data available at http://www.worldclim.org/)^35^. Brown bears enter and exit the den at a mean temperature of approximately 0 °C^36^. Therefore, we calculated the mean temperature of the wintering season (*T*_*ws*_) as the mean temperature across the months, in which the maximum monthly temperature was below 0 °C. Moreover, we estimated net primary productivity (*NPP*; g dry matter m^−2^ a^−1^), based on the minimum of the temperature- and precipitation-limited rates of *NPP* using the Miami model^37^. The two limiting rates were calculated independently as simple empirical functions of mean annual temperature (*T*) and total annual precipitation (*P*), following the equations given in Adams *et al*.^37^:

1 $$\begin{array}{c}NPP=\,{\rm{\min }}(NP{P}_{T},\,NP{P}_{P}),\,{\rm{with}}\,\\ NP{P}_{T}=3000{(1+\exp (1.315-0.119T))}^{-1}\,{\rm{and}}\\ NP{P}_{P}=3000(1-\exp (-0.000664P)).\end{array}$$

The model does not treat the effects of seasonality, CO_2_, humidity, light capture, or vegetation and soil types explicitly^37^. However, we chose this method, because it allowed for a direct comparison with the results of the metapopulation model. Given the lack of detailed data about *NPP* during the Holocene, the Miami model provides a good first-order characterization of the major climatic controls on ecosystem productivity during the past 12,000 years.

We fitted a generalized linear mixed effects model to analyse variation in reproductive rate using a *gamma* distribution with an *identity*-link function. In this model, we included a random effect for continent (North America, Europe and Asia) to account for differences in the phylogeographic histories of brown bear populations across continents^38–40^. Moreover, we included female body mass as a fixed explanatory variable to account for local adaptations to differences in environmental conditions among the brown bear populations^41, 42^. We included *NPP* and *T*_*ws*_ into the model to test the hypothesis that energy availability during the growing season and energy expenditure during the wintering season constrain the reproductive rate of the brown bear. As the sample size varied among the study populations, we performed the analysis with and without weighting of the observations by their sample size. We calculated the weight of observations as *w* = log_10_(*N*_*FM*_) + log_10_(*N*_*LI*_) + log_10_(*N*_*LS*_), where *N*_*FM*_, *N*_*LI*_ and *N*_*LS*_ were the sample sizes for female body mass, inter-birth interval and litter size, respectively. In the case of observations with missing information about sample size, we assumed a sample size of one. Weighted and non-weighted analyses led to identical conclusions. In the main text we report results of the weighted analysis, because it incorporates variability in uncertainty in the estimates of life-history traits among populations. We assessed whether the analysis was affected by collinearity among predictors, by calculating variance inflation factors and by using alternative variables to characterize the growing season and found the results of our analysis to be robust (Supplementary Methods and Supplementary Table 1).

### Metapopulation model

To estimate the spatiotemporal population dynamics of the European brown bear during the Holocene, we fitted a Bayesian hierarchical metapopulation model^24, 43^ to detection/non-detection data based on archaeofaunal and recent occurrence records. Below we give detailed information about the spatial and temporal resolution of the analysis, the data used, and the structure of the model.

### Spatial and temporal resolution of analysis

For our analysis, we defined seven periods: (1) 10000–7000 BCE, (2) 7000–3000 BCE, (3) 3000 BCE – 0 CE, (4) 0–1500 CE, (5) 1500–1800 CE, (6) 1950–1970 CE, and (7) 2010–2015 CE. We used the midpoint of each of the seven periods to determine the length of the time intervals Δ*t* between the respective periods in the metapopulation model (e.g., Δ*t*_12_ = 8500–5000 = 3500 years). The time intervals Δ*t* between periods were 3500, 3500, 2250, 900, 310, and 52.5 years, respectively. Moreover, we used a 100 × 100 km^2^ grid (100-km grid, hereafter) including 789 terrestrial cells across Europe for the analysis (Fig. 2c). By using a coarse grain of 100 km in our analysis, we minimized the problem of missing data in the archeofaunal occurrence records and avoided inflating data quality due to inappropriate downscaling of reconstructed past land use and climate data^44^.

### Occurrence data

For the first five periods (covering 10000 BCE – 1800 CE) we compiled spatially explicit detection histories of the brown bear across Europe using a database containing published and unpublished archeofaunal records from dated archaeological and paleontological excavation sites^19, 25–27^ (Supplementary Methods and Supplementary Fig. 2). The excavation sites included settlements (*n* = 2,735), castles (*n* = 371), caves (*n* = 274), burial sites (*n* = 264), middens and rubbish pits (*n* = 174), cult and religious sites (*n* = 76), moors and riverbeds (*n* = 11), as well as 272 sites without known designation. For each record, we extracted data on the presence and abundance of subfossil bone remains of brown bear, red deer (*Cervus elaphus*), and roe deer (*Capreolus capreolus*) (total abundance, median: 7, range: 1–26,428). Records containing red or roe deer, but not brown bear, served as negative controls, because both deer species were typical elements of the European fauna during the Holocene and the most frequent prey of European Stone Age hunters^25, 26^. The rationale behind this approach is that if remains of red or roe deer were present at a given site, it was suitable for preservation of bone material and, thus, for detection of brown bears, if the species had been present. This allowed us to account for imperfect detection in the archaeofaunal record, while simultaneously modelling the spatiotemporal variation in brown bear extinction and colonization rates across the European continent. In total, our dataset comprised 4,177 archeofaunal records from 3,461 excavation sites (records from archaeological layers with distinct temporal context were treated separately). We used the above records of brown bear *presence* and *absence* in each cell and period to obtain detection/non-detection data (i.e., detection histories) for the first five periods (i.e., 10000 BCE – 1800 CE). To inform the model about the distribution of the brown bear in the last two periods, we used recent occurrence data from Chapron *et al*.^20^ and Chestin^45^ (Supplementary Fig. 2). These data were resampled to the 100-km grid.

### Land-use data

We compiled land-use data from the *HYDE 3.1* database^46^. These data represent spatially explicit reconstructions of fractional land use covering the period 10000 BCE to 2000 CE. The data have a spatial resolution of 5′ × 5′ and a temporal resolution of 1,000-year time intervals for the BCE period, then 100-year time intervals until 1700 CE, and 10-year time intervals until 2000 CE. For analysis, we extracted three variables reflecting human impact on the environment, namely the proportion of a given cell covered by agriculture, pastures, and urban areas. We used the sum of these variables as a proxy for human land use, that is, the total fraction of land appropriated by humans. Then we averaged the fractional land use for each cell across the time intervals that fell in each of the seven periods used for the metapopulation model. The time intervals that occurred at the margins of the periods received only half the weight in the calculations compared to those time intervals in the core of the periods. We resampled the land-use data to the 100-km grid for analysis. To account for uncertainties due to different assumptions about historical changes in land-use intensification (i.e., per capita land-use intensity)^47, 48^, we considered two different scenarios of the *HYDE 3.1* database in our analysis (Supplementary Methods and Supplementary Fig. 4).

We decided to use land use as a proxy for human impact, because unlike other measures, such as human population density, land use integrates the various effects of humans on brown bear populations. First, previous work has shown that habitat loss due to land use severely constrains the occurrence and breeding range of the European brown bear^49, 50^. Second, the potential for human-bear conflicts due to predation on livestock or wild ungulates increases in intensively used landscapes^51^. Therefore, land-use intensification historically coincided with the persecution and local extirpation of the brown bear and other large carnivores in Europe^21, 52^. This is why persecution and habitat loss are generally the most important drivers of extinction risk in mammals, and particularly in large carnivores^22, 53^. While the behaviour of humans in relation to bears might well have changed through time, there is evidence that humans have hunted bears since at least 9000 BP^54^ and that bear hunting and the associated rituals and beliefs were deeply rooted in the prehistoric culture of humans in the northern hemisphere^55^. Thus, we expect land use to be a reliable proxy of the combined effects of hunting, persecution and human-induced habitat loss.

### Climate, net primary productivity and elevation data

We compiled climate data from Luterbacher *et al*.^56^, Pauling *et al*.^57^, and Mauri *et al*.^30^. The data from Luterbacher *et al*.^56^ and Pauling *et al*.^57^ represent spatially explicit seasonal temperature and precipitation reconstructions covering the period 1500–2000 CE. The data have a spatial resolution of 0.5° × 0.5° and a temporal resolution of 1-year. The data from Mauri *et al*.^30^ represent spatially explicit pollen-based climate reconstructions covering the period 12000 BP (before present) to 100 BP. The reconstructions are given as anomalies relative to late preindustrial time, centred approximately around 100 BP (i.e., 1850 CE), with a dating uncertainty of ± 250 years^30^. The data have a spatial resolution of 1° × 1° and a temporal resolution of 1,000-year time intervals until 1000 BP and a single 900-year time interval until 100 BP, respectively. From these datasets, we extracted mean annual temperature, total annual precipitation (to estimate *NPP* see below) and mean winter temperature for analysis.

We first calculated 31-year running averages for each of the three variables from Luterbacher *et al*.^56^ and Pauling *et al*.^57^ for each cell for the period 1500 to 2000 CE. Then we used the 31-year running averages for the year 1850 CE as the baseline to calibrate the anomalies reported in Mauri *et al*.^30^. We estimated net primary productivity (g dry matter m^−2^ a^−1^) from mean annual temperature and total annual precipitation using the Miami model^37, 58, 59^ in equation (1). For analysis, we used the same time intervals and weighting scheme as for the land-use data to calculate mean net primary productivity and mean winter temperature for each cell of the 100-km grid in each of the seven periods.

We extracted high-resolution elevation data from WorldClim^35^ using an initial resolution of 0.5′ × 0.5′ (~1 km^2^ at the equator). For analysis, we resampled the elevation data to the 100-km grid.

### Model structure

To decompose the contribution of humans and climate change to the Holocene decline of the European brown bear, we combined a spatially explicit metapopulation model with a path analysis in a Bayesian hierarchical framework^24, 60^. In particular, we based our analysis on a hierarchical state-space formulation of a spatially explicit metapopulation model that accounts for imperfect detection of brown bear occurrence in the archeofaunal record^24^. To describe detection/non-detection data *y*_*s,t*_ in grid cell *s* in period *t*, the hierarchical state-space model is based on two linked stochastic processes^24^: a sub-model for the observations conditional on the unobserved state process, i.e., *y*_*s,t*_ | *z*_*s,t*_ (*observation model*) and a sub-model for the unobserved or partially observed state process *z*_*s,t*_ (*state model*).

### State model

The *state model* has a simple formulation in terms of initial occurrence probability, i.e., at *t* = 1, which we will designate *μ*_*z,s,t* = 1_. The initial occurrence states are assumed to be *independent identically distributed* Bernoulli random variables, denoted as z_*s*,*t* = 1_ ~ Bernoulli(*μ*_*z,s,t* = 1_). In all subsequent periods, the occurrence state *z*_*s,t* + 1_ is a Bernoulli trial with a success parameter *μ*_*z,s,t* + 1_ that depends on whether cell *s* was occupied in period *t* and on the value of two parameters that indicate either the probability of extinction, *p*_*φ*_, or colonization, *p*_*γ*_, so that:

2 $$\begin{array}{c}{\mu }_{z,s,t+1}={z}_{s,t}(1-{p}_{\phi ,s,t})+(1-{z}_{s,t}){p}_{\gamma ,s,t}\,{\rm{with}}\\ {z}_{s,t+1}|{z}_{s,t} \sim {\rm{Bernoulli}}({\mu }_{z,s,t+1}).\end{array}$$

Because the lengths of the periods Δ*t* in our dataset were unequal (i.e., they ranged from 3500 to 52.5 years), the assumptions of the discrete-time Markov process were violated^61^, preventing direct estimation of transition probabilities from our data. Therefore, we used Kolmogorov’s forward equations^61^ (equivalent to matrix exponentiation) to map the colonization (*r*_*γ*_) and extinction (*r*_*φ*_) rates for each time interval Δ*t* on the colonization (*p*_*γ*_) and extinction (*p*_*φ*_) probabilities, respectively:

3 $${p}_{\gamma ,s,t}=1-({r}_{\phi ,s,t}+{r}_{\gamma ,s,t}\exp (-({r}_{\phi ,s,t}+{r}_{\gamma ,s,t}){\rm{\Delta }}t))/({r}_{\phi ,s,t}+{r}_{\gamma ,s,t}),$$

4 $${p}_{\phi ,s,t}=1-({r}_{\gamma ,s,t}+{r}_{\phi ,s,t}\exp (-({r}_{\phi ,s,t}+{r}_{\gamma ,s,t}){\rm{\Delta }}t))/({r}_{\phi ,s,t}+{r}_{\gamma ,s,t}),$$

We modelled the extinction rate (*r*_*φ,s,t*_) as a function of land use (*LU*), winter temperature (*WT*), net primary productivity (*NPP*), and elevation (*ELE*) with a *logarithmic*-link function:

5 $$\mathrm{log}({r}_{\phi ,s,t})={\beta }_{\phi ,0}+{\beta }_{\phi ,LU}L{U}_{s,t}+{\beta }_{\phi ,NPP}NP{P}_{s,t}+{\beta }_{\phi ,WT}W{T}_{s,t}+{\beta }_{\phi ,ELE}EL{E}_{s,t},$$

We modelled the colonization rate *r*_*γ,s,t*_ of the species as a function of spatial connectivity to extant cells with a *logarithmic*-link function. Connectivity describes the distance-dependent influence of all potential neighbouring source populations *s*' via a negative exponential dispersal kernel^62^:

6 $$\mathrm{log}({r}_{\gamma ,s,t})=\lambda \sum _{s\ne {s}^{^{\prime} }}\exp (-\alpha {d}_{s,{s}^{^{\prime} }}){z}_{{s}^{^{\prime} },t}$$

where *z*_*s′,t*_ is the occupancy of cell *s′* in period *t*, *d*_*s,s′*_ is the distance between cells *s* and *s′*, 1⁄*α* is the average dispersal distance, and *λ* is a constant. We did not estimate the additional scaling constant *λ* from our data^62^, because direct estimation of this parameter from our data would cause parameter redundancy with the intercept *β*_*φ*,0_ in equation (5). To avoid problems with parameter identifiability, we estimated *β*_*φ*,0_ and set *λ* = 1^43^. Using alternative values of *λ* did not affect any of our conclusions, because it causes a proportional shift of the estimate for the intercept *β*_*φ*,0_. To account for coastline shape and elevation differences (terrain shape), we calculated dispersal distances *d* between cells as Least Cost Path (LCP) distances (Supplementary Methods). We extracted high-resolution elevation data from WorldClim^35^ (0.5′ × 0.5′ resolution) and resampled these data to a resolution of 5 × 5 km^2^ for calculation of Least Cost Path (LCP) distances. To account for uncertainty regarding colonization of the European continent by the brown bear from Asia^19, 38, 63^ we considered two scenarios with and without colonization of Europe from Asia, respectively (Supplementary Methods and Supplementary Fig. 5).

### Observation model

We linked the latent occurrence state *z*_*s,t*_ of the *state model* through the *observation model* to each occurrence record *i* associated with cell *s* in period *t*, *y*_*i,s*[*i*]*,t*[*i*]_. Therefore, we specified the *observation model* conditional on the latent process *z*_*s,t*_ as:

7 $${y}_{i,s[i],t[i]}|{z}_{s,t} \sim {\rm{Bernoulli}}({z}_{s,t}{p}_{i,s[i],t[i]}),$$

Thus, if cell *s* is occupied in period *t*, the data are Bernoulli trials with a parameter for the detection probability, *p*_*i,s*[*i*]*,t*[*i*]_. If cell *s* is unoccupied in period *t*, then the data are Bernoulli trials with *P*(*y*_*s,t*_ = 1) = 0. For the first five periods that were informed by archeofaunal records, we modelled the detection probability *p*_*i,s*[*i*]*,t*[*i*]_ associated with the *i*th record as a function of the total number of bone remains (*NSP*) of the three species in a given excavation record and the type of excavation site (*TYP*) from which the records originated using a *logit*-link function as:

8 $${\rm{logit}}({p}_{i,s[i],t[i]})={\beta }_{p,0}+{\beta }_{p,NSP}\,\mathrm{log}(NS{P}_{i})+{\beta }_{p,TYP}TY{P}_{i},$$

The number of bone remains reflects both sampling effort and the potential of an excavation site to preserve bone material^4, 28^ and also may reflect the intensity of hunting activities and thus the hunting pressure on the regional fauna^64^. The type of excavation site may reflect cultural habits that could affect the prevalence of brown bear remains in certain types of excavation sites (e.g., burial sites associated with rituals)^65^. For the 272 of the 4,177 excavation records for which we did not have information about the designation of the site, we randomly assigned a site type *TYP*_*i*_ based on the relative occurrence of the seven site types in the different periods following a multinomial distribution: *TYP*_*i,s*[*i*]*,t*[*i*]_ ~ Multinomial(**P**_*n*,*t*[*i*]_), where **P** is a matrix with *n* rows indicating the seven site types and *t* columns indicating the five periods for which we had archeofaunal records, and each cell contains the frequency of occurrence of each site type in each period. Therefore, the uncertainty originating from these records was fully propagated through all steps of model estimation. For the last two periods (i.e., 1950–70 s and recent) that were informed by species distribution maps, we assumed perfect detection, so that *y*_*i,s*[*i*]*,t*[*i*]_ = *z*_*s,t*_. We are confident that this is a reasonable assumption, given the coarse grain of our analysis (i.e., 100 × 100 km) and the huge effort spent in the monitoring of European brown bear populations since the 1950s^20^.

### Path analysis

Besides this state-space model describing the direct effects of the explanatory variables on the extinction rate, the path model contained three regression equations to describe the potential causal relationships among the predictor variables. To account for spatial autocorrelation, these regressions included a random effect for grid cell. We modelled the effect of elevation on winter temperature as:

9 $$\begin{array}{c}{\mu }_{WT,s,t}={\beta }_{WT,0}+{\beta }_{WT,ELE}EL{E}_{s,t}+cel{l}_{WT,s},\,{\rm{with}}\\ W{T}_{s,t} \sim {\rm{Normal}}({\mu }_{WT,s,t},{\tau }_{WT}),{\rm{and}}\,cel{l}_{WT,s} \sim {\rm{Normal}}(0,{\tau }_{WT,cell}).\end{array}$$

We modelled the effect of winter temperature and elevation on net primary productivity as:

10 $$\begin{array}{c}{\mu }_{NPP,s,t}={\beta }_{NPP,0}+{\beta }_{NPP,WT}W{T}_{s,t}+{\beta }_{NPP,ELE}EL{E}_{s,t}+cel{l}_{NPP,s},\,{\rm{with}}\\ NP{P}_{s,t} \sim {\rm{Normal}}({\mu }_{NPP,s,t},{\tau }_{NPP})\,{\rm{and}}\,cel{l}_{NPP,s} \sim {\rm{Normal}}(0,{\tau }_{NPP,cell}).\end{array}$$

Finally, we modelled the effect of the aforementioned variables on human land use as:

11 $$\begin{array}{c}{\mu }_{LU,s,t}={\beta }_{LU,0}+{\beta }_{LU,NPP}NP{P}_{s,t}+{\beta }_{LU,WT}W{T}_{s,t}+{\beta }_{LU,ELE}EL{E}_{s,t}+cel{l}_{LU,s},\,{\rm{with}}\\ L{U}_{s,t} \sim {\rm{Normal}}({\mu }_{LU,s,t},{\tau }_{LU})\,{\rm{and}}\,cel{l}_{LU,s} \sim {\rm{Normal}}(0,{\tau }_{LU,cell}).\end{array}$$

### Model selection

To select informative paths, we used *Stochastic Search Variable Selection* (SSVS) with global adaptation^66^. The variable selection procedure can be seen as one of deciding which of the regression parameters *β*_*j*_ in a given model are equal to zero. To denote whether the variable *j* is present in the model, we used an indicator variable *I*_*j*_ (where *I*_*j*_ = 1 indicates presence, and *I*_*j*_ = 0 absence of variable *j* in the model). The prior inclusion probability of all explanatory variables was set to *P* = 0.5. The SSVS approach uses a mixture prior for each regression parameter *β*_*j*_ following the equation:

12 $$P({\beta }_{j}|{I}_{j})=(1-{I}_{j})\cdot {\rm{Normal}}(0,{\tau }_{\beta })+{I}_{j}\cdot {\rm{Normal}}(0,g{\tau }_{\beta }),$$

where the first density (the spike) is centred around zero and has a small variance^66^. Here we use a random effects variant of SSVS^66^, in which in the spike part of the prior *τ*_*β*_ is fixed to a constant (*τ*_*β*_ = 3,600), and in the slab part of the prior the product g*τ*_*β*_ is estimated by the model. The variance *σ* ^2^_*β*_ = g*τ*_*β*_^−0.5^ was drawn from a uniform prior between 0 and 20. This form of global adaptation has the advantage of facilitating the tuning of the variable selection, because the distribution of each coefficient is shrunk towards the correct region of the parameter space by the other coefficients in the model. In order to infer which of the variables should be included in the models, we used Bayes factors^66^.

### Model implementation

The models were fitted using an Markov Chain Monte Carlo (MCMC) algorithm with a Gibbs sampler in *JAGS* ^67^ called from *R* ^68^ by using package *rjags* ^69^ (for the *JAGS* code see Supplementary Computer Code 1). We used uninformative prior distributions for most parameters. Running the models with a uniform prior for the dispersal parameter *α* suggested that the information contained in our dataset did not suffice to inform this parameter, because the estimate for this parameter was poorly defined. Therefore, we specified an informative prior following a gamma distribution (shape = 3, rate = 1) with highest mass around 2 for the dispersal parameter *α*, which corresponds to an average dispersal distance of 1⁄*α* = 50 km (given the 100-km grid). This value roughly corresponds to the average natal dispersal distance for Scandinavian brown bears (males: 108.3 km ± 27.4 km; females: 15.7 km ± 2.4 km)^70^. The decision of whether to use an informative or uninformative prior for the dispersal parameter did not affect any of the results or conclusions.

We ran 10 parallel chains for each model. The initial values for the chains were drawn randomly from uniform distributions. The models were run for 4,500 iterations with an adaptive burn-in phase of 2,000 iterations and a thinning interval of 25 iterations, resulting in 100 samples per chain, corresponding to 1,000 samples from the posterior distribution. The chains were checked for convergence, temporal autocorrelation, and effective sample size using the *R* package *coda* ^71^. In the main text, we report the parameter estimates after pooling the posterior samples from the two-factorial design with two scenarios for changes in per capita land-use intensity during the Holocene (constant *versus* decreasing) and two scenarios for colonization of the European continent by the brown bear from Asia (yes *versus* no), respectively (Supplementary Methods and Supplementary Figs 4 and 5). Therefore, these estimates account for potential biases due to the uncertainty in these model assumptions. The models were run on the high-performance computing cluster of the PL-Grid Infrastructure.

### Data availability

The datasets generated and analysed during the current study are available from the corresponding author upon reasonable request.

## Electronic supplementary material

Supplementary Information

## References

[1] Alroy J (2001). A multispecies overkill simulation of the end-Pleistocene megafaunal mass extinction. Science.

[2] Stuart AJ, Kosintsev PA, Higham TFG, Lister AM (2004). Pleistocene to Holocene extinction dynamics in giant deer and woolly mammoth. Nature.

[3] Lorenzen ED (2011). Species-specific responses of Late Quaternary megafauna to climate and humans. Nature.

[4] Duncan RP, Boyer AG, Blackburn TM (2013). Magnitude and variation of prehistoric bird extinctions in the Pacific. Proc. Natl. Acad. Sci. USA.

[5] Allentoft ME (2014). Extinct New Zealand megafauna were not in decline before human colonization. Proc. Natl. Acad. Sci. USA.

[6] Cooper A (2015). Abrupt warming events drove Late Pleistocene Holarctic megafaunal turnover. Science.

[7] Crees JJ (2016). Millennial-scale faunal record reveals differential resilience of European large mammals to human impacts across the Holocene. Proc. R. Soc. London, Ser. B..

[8] Guthrie RD (2003). Rapid body size decline in Alaskan Pleistocene horses before extinction. Nature.

[9] Sandom C, Faurby S, Sandel B, Svenning J-C (2014). Global late Quaternary megafauna extinctions linked to humans, not climate change. Proc. Biol. Sci..

[10] Barnosky AD, Koch PL, Feranec RS, Wing SL, Shabel AB (2004). Assessing the Causes of Late Pleistocene Extinctions on the Continents. Science.

[11] Koch PL, Barnosky AD (2006). Late Quaternary Extinctions: State of the Debate. Annu. Rev. Ecol. Evol. Syst..

[12] Brook BW, Sodhi NS, Bradshaw CJA (2008). Synergies among extinction drivers under global change. Trends Ecol. Evol..

[13] Büntgen U (2011). 2500 years of European climate variability and human susceptibility. Science.

[14] Eriksson A (2012). Late Pleistocene climate change and the global expansion of anatomically modern humans. Proc. Natl. Acad. Sci. USA.

[15] Tallavaara M, Luoto M, Korhonen N, Järvinen H, Seppä H (2015). Human population dynamics in Europe over the Last Glacial Maximum. Proc. Natl. Acad. Sci. USA.

[16] Humphries MM, Thomas DW, Speakman JR (2002). Climate-mediated energetic constraints on the distribution of hibernating mammals. Nature.

[17] Davidson AD, Hamilton MJ, Boyer AG, Brown JH, Ceballos G (2009). Multiple ecological pathways to extinction in mammals. Proc. Natl. Acad. Sci. USA.

[18] Fritz SA (2013). Diversity in time and space: wanted dead and alive. Trends Ecol. Evol..

[19] Sommer RS, Benecke N (2005). The recolonization of Europe by brown bears Ursus arctos Linnaeus, 1758 after the Last Glacial Maximum. Mamm. Rev..

[20] Chapron G (2014). Recovery of large carnivores in Europe’s modern human-dominated landscapes. Science.

[21] Zedrosser A, Steyaert SMJG, Gossow H, Swenson JE (2011). Brown bear conservation and the ghost of persecution past. Biol. Conserv..

[22] Wolf C, Ripple WJ (2017). Range contractions of the world’s large carnivores. R. Soc. Open Sci..

[23] Robbins CT, Ben-David M, Fortin JK, Nelson OL (2012). Maternal condition determines birth date and growth of newborn bear cubs. J. Mammal..

[24] Royle JA, Kéry M (2007). A bayesian state-space formulation of dynamic occupancy models. Ecology.

[25] Sommer RS (2008). Late Quaternary distribution dynamics and phylogeography of the red deer (Cervus elaphus) in Europe. Quat. Sci. Rev..

[26] Sommer RS, Fahlke JM, Schmölcke U, Benecke N, Zachos FE (2009). Quaternary history of the European roe deer Capreolus capreolus. Mamm. Rev..

[27] Benecke, N., von den Driesch, A. & Heinrich, D. *Holozängeschichte der Tierwelt Europas*., doi:10.13149/001.mcus7z-2 (Datensammlung hrsg. v. IANUS, 2016).

[28] Turvey ST, Blackburn TM (2011). Determinants of species abundance in the Quaternary vertebrate fossil record. Paleobiology.

[29] Nogués-Bravo D, Araújo MB, Romdal T, Rahbek C (2008). Scale effects and human impact on the elevational species richness gradients. Nature.

[30] Mauri A, Davis BAS, Collins PM, Kaplan JO (2015). The climate of Europe during the Holocene: a gridded pollen-based reconstruction and its multi-proxy evaluation. Quat. Sci. Rev..

[31] Green ACMC (1996). Did the Romans Hunt?. Class. Antiq..

[32] Matheson C (1942). Man and Bear in Europe. Antiquity.

[33] Kaplan JO, Krumhardt KM, Zimmermann N (2009). The prehistoric and preindustrial deforestation of Europe. Quat. Sci. Rev..

[34] Stearns, S. *The Evolution of Life Histories*. (Oxford University Press, 1992).

[35] Hijmans RJ, Cameron SE, Parra JL, Jones PG, Jarvis A (2005). Very high resolution interpolated climate surfaces for global land areas. Int. J. Climatol..

[36] Evans AL (2016). Drivers of hibernation in the brown bear. Front. Zool..

[37] Adams B, White A, Lenton TM (2004). An analysis of some diverse approaches to modelling terrestrial net primary productivity. Ecol. Modell..

[38] Davison J (2011). Late-Quaternary biogeographic scenarios for the brown bear (Ursus arctos), a wild mammal model species. Quat. Sci. Rev..

[39] Keis M (2013). Complete mitochondrial genomes and a novel spatial genetic method reveal cryptic phylogeographical structure and migration patterns among brown bears in north-western Eurasia. J. Biogeogr..

[40] Leonard JA, Wayne RK, Cooper A (2000). Population genetics of ice age brown bears. Proc. Natl. Acad. Sci. USA.

[41] Ferguson SH, Mcloughlin PD (2000). Effect of energy availability, seasonality, and geographic range on brown bear life history. Ecography.

[42] Mcloughlin PD, Ferguson SH, Messier F (2000). Intraspecific variation in home range overlap with habitat quality: A comparison among brown bear populations. Evol. Ecol..

[43] Kery, M. & Schaub, M. *Bayesian Population Analysis using WinBUGS*. (Academic Press, 2012).

[44] Hurlbert AH, Jetz W (2007). Species richness, hotspots, and the scale dependence of range maps in ecology and conservation. Proc. Natl. Acad. Sci. USA.

[45] Chestin IE (1997). Dynamics of brown bear range and status of isolated populations in European Russia, Western Siberia and adjacent countries. Int. Conf. Bear Res. Manag..

[46] Klein Goldewijk K, Beusen A, Van Drecht G, De Vos M (2011). The HYDE 3.1 spatially explicit database of human-induced global land-use change over the past 12,000 years. Glob. Ecol. Biogeogr..

[47] Klein Goldewijk K, Verburg PH (2013). Uncertainties in global-scale reconstructions of historical land use: An illustration using the HYDE data set. Landsc. Ecol..

[48] Ellis EC (2013). Used planet: a global history. Proc. Natl. Acad. Sci. USA.

[49] Naves J, Wiegand T, Revilla E, Delibes M (2003). Endangered Species Constrained by Natural and Human Factors: the Case of Brown Bears in Northern Spain. Conserv. Biol..

[50] Fernández N, Selva N, Yuste C, Okarma H, Jakubiec Z (2012). Brown bears at the edge: Modeling habitat constrains at the periphery of the Carpathian population. Biol. Conserv..

[51] Bautista, C. *et al*. Patterns and correlates of claims for brown bear damage on a continental scale. *J. Appl. Ecol*. doi:10.1111/1365-2664.12708 (2016).

[52] Breitenmoser U (1998). Large predators in the Alps: The fall and rise of man’s competitors. Biol. Conserv..

[53] Revilla E, González-Suárez M (2014). Generalized drivers in the mammalian endangerment process. PLoS One.

[54] Helskog K (2012). Bears and meanings among hunter-fisher-gatherers in Northern Fennoscandia 9000 – 2500 BC. Cambridge Archaeol. J..

[55] Hallowell AI (1926). Bear ceremonialism in the northern hemisphere. Am. Anthropol..

[56] Luterbacher J, Dietrich D, Xoplaki E, Grosjean M, Wanner H (2004). European seasonal and annual temperature variability, trends, and extremes since 1500. Science.

[57] Pauling A, Luterbacher J, Casty C, Wanner H (2005). Five hundred years of gridded high-resolution precipitation reconstructions over Europe and the connection to large-scale circulation. Clim. Dyn..

[58] Zaks DPM, Ramankutty N, Barford CC, Foley JA (2007). From Miami to Madison: Investigating the relationship between climate and terrestrial net primary production. Global Biogeochem. Cycles.

[59] Del Grosso S (2008). Global potential net primary production predicted from vegetation class, precipitation, and temperature. Ecology.

[60] Clough Y (2012). A generalized approach to modeling and estimating indirect effects in ecology. Ecology.

[61] Welton N, Ades A (2005). Estimation of Markov Chain Transition Probabilities and Rates from Fully and Partially Observed Data: Uncertainty Propagation, Evidence Synthesis, and Model Calibration. Med. Decis. Mak..

[62] Hanski I, Ovaskainen O (2000). The metapopulation capacity of a fragmented landscape. Nature.

[63] Kopatz A (2014). Admixture and gene flow from Russia in the recovering Northern European brown bear (Ursus arctos). PLoS One.

[64] Broughton, J. M. Prehistoric Human Impacts on California Birds: Evidence from the Emeryville Shellmound Avifauna. *Ornithol. Monogr*. 1–90 (2004).

[65] Iregren, E. Lappish Bear Graves in Northern Sweden: An Archaeological and Osteological Study. in *Early Norrland* **5**, 113 (Kungl. Vitterhets Historie och Antikvitets Akademien, 1974).

[66] O’Hara RB, Sillanpää MJ (2009). A review of bayesian variable selection methods: What, how and which. Bayesian Anal..

[67] Plummer, M. JAGS: A program for analysis of Bayesian graphical models using Gibbs sampling. (2003).

[68] R Development Core Team. R: A language and environment for statistical computing. R Foundation for Statistical Computing, Vienna, Austria. Available at: https://www.R-project.org/ (2016).

[69] Plummer, M. rjags: Bayesian Graphical Models using MCMC. (2015).

[70] Støen O-G, Zedrosser A, Sæbø S, Swenson JE (2006). Inversely density-dependent natal dispersal in brown bears Ursus arctos. Oecologia.

[71] Plummer M, Best N, Cowles K, Vines K (2006). CODA: convergence diagnosis and output analysis for MCMC. R News.

[72] South A (2011). rworldmap: A New R package for Mapping Global Data. R J..

[73] South, A. rworldxtra: Country boundaries at high resolution. R package version 1.01. https://CRAN.R-project.org/package=rworldxtra. (2012).

