# Supplementary material for: Humans and climate change drove the Holocene decline of the brown bear
Source: Sci Rep. 2017 Sep 4;7:10399. doi: 10.1038/s41598-017-10772-6 (PMC5583342; doi:10.1038/s41598-017-10772-6)
Supplement: Supplementary file 1 — Supplementary Information [file 41598_2017_10772_MOESM1_ESM.pdf]

Supplementary Information for

"Humans and climate change drove the Holocene decline of the brown bear"

Jörg Albrecht[1,*,§], Kamil A. Bartoń[1], Nuria Selva[1], Robert S. Sommer[2], Jon E. Swenson[3,4], Richard Bischof[3]

[1]Institute of Nature Conservation, Polish Academy of Sciences, Mickiewicza 33, PL-31-120 Kraków, Poland.

[2]Department of Zoology, Institute of Bioscience, University of Rostock, Universitätsplatz 2, D-18055 Rostock, Germany.

[3]Faculty of Environmental Sciences and Natural Resource Management, Norwegian University of Life Sciences, PO Box 5003, NO-1432 Ås, Norway

[4]Norwegian Institute for Nature Research, NO-7485 Trondheim, Norway.

*Correspondence and requests for materials should be addressed to J.A. (email: joerg.albrecht@senckenberg.de)

[§]Present address: Senckenberg Biodiversity and Climate Research Centre (BiK-F), Senckenberganlage 25, 60325 Frankfurt am Main, Germany.

Supplementary Methods

Evaluation of potential collinearity problems in the meta-analysis. To evaluate potential collinearity problems that may arise from linear relationships between model covariates, we calculated variance inflation factors (VIFs)[1]. VIFs for all covariates were lower than 8.16, less than the threshold of 10 above which collinearity may adversely affect regression results[1] (Supplementary Table 1). To further assess how robust our analysis was with respect to collinearity among predictors, we calculated two alternative variables to characterize the growing season. First, we used growing degree-days (*GDD*; base 5 °C; data available at http://www.sage.wisc.edu/atlas/)[2], as a proxy for the length of the growing season, because the length of the growing season is indirectly related to net primary productivity via plant growth[3]. Second, we used the mean monthly temperature of the growing season (T_{gs}), because, similar to the length of the growing season, the mean temperature of the growing season is indirectly related to net primary productivity via plant growth[3]. We calculated T_{gs} as the mean temperature across the months, in which the minimum monthly temperature was above 5 °C. We used a threshold of 5 °C for the growing season to be consistent with the base temperature for growing degree-days. Using these alternative variables in the analysis reduced VIFs from 8 to 3 but led to identical conclusions, suggesting that collinearity does not affect the conclusions from this analysis (Supplementary Table 1).

Uncertainty in assumptions about Holocene changes in land-use intensification and colonization of Europe by the brown bear from Asia. To account for uncertainties due to different assumptions about historical changes in land-use intensification (i.e., per capita land-use intensity)[4,5], we considered two different scenarios in our analysis (Supplementary Fig. 4). The first scenario (*HYDE 3.1 baseline*) effectively omitted land-use intensification by assuming that per capita land-use remained approximately constant over time and that the proportion of land used for agriculture, pastures and urban areas increased linearly with

increasing population density. In this scenario, land-use intensity closely resembled the pattern in 1961 CE, the first year for which global Food and Agriculture Organization of the United Nations statistics are available[4–6]. The second scenario (*HYDE 3.1 concave*) assumed a non-linear, concave relationship between population density and per capita land use through time. That is, low-density populations with high per capita land use first expanded to fill all usable land and then intensified land use (i.e., use less land per capita) as population densities increased over time[4,5]. This assumption is similar to the assumption of the *KK10* model[7,8]. The global estimates of land use for the early Holocene from the *HYDE 3.1 concave* scenario and the *KK10* model[4,7] closely resembled each other and provided an upper-bound estimate of land use in the early Holocene, whereas the *HYDE 3.1 baseline* scenario provided a lower-bound estimate of land use during the same period.

To account for uncertainty regarding colonization of the European continent by the brown bear from Asia[9–11] we considered two scenarios. The first scenario assumed that there was no permanent source population at the eastern border of the study area, while the second scenario allowed for colonization from Asia from a permanent source population at the eastern border. To implement these two scenarios, we fixed the state of the eastern most cells in the occupancy matrix $Z_{s,t}$ in the second scenario to permanently occupied, i.e., we set $z_{s,t} = 1$, whereas these cells were not informed *a priori* in the first scenario, so that the occupancy state of these cells was estimated by the model (Supplementary Fig. 5).

In the main text we report the parameter estimates after pooling the posterior samples from the two-factorial design with two scenarios for changes in per capita land-use intensity during the Holocene (constant *versus* decreasing) and two scenarios and two scenarios for colonization of the European continent by the brown bear from Asia (yes *versus* no), respectively.

Compilation of the archaeofaunal database. Archaeofaunal records were obtained from the Holocene vertebrate database[9,12–14]. In most cases, subfossil bones were context dated from their assignment to an archaeological layer. These dates were often radiocarbon supported and obtained from other materials (e.g., bones or charcoal) from the layer in which subfossil bones were recovered[9,12,13,15]. In a minority of cases (<1% of records) the absolute age of subfossil bones was available from ^{14}C dating (AMS dating and conventional radiocarbon dating). In the few cases where absolute dates were available these were preferred over relative dates, because relative dates are generally less precise and can sometimes be erroneous due to complicated stratigraphy[14].

In addition, we applied the following protocol to all records of the archeofaunal database: All archaeofaunal records were mapped in Google Earth (https://earth.google.de/) to validate location information from the database and correct assignment of geographic coordinates (latitude, longitude). Where location information was incorrect, the correct site or location was searched for to obtain accurate geographic coordinate information. Where information for records was missing, the original reference was searched and information filled in. If crucial information on the date or location of the record could not be found, it was rejected.

Implementation of least cost path distances in the metapopulation model. To account for coastline shape and elevation differences (terrain shape), we calculated dispersal distances *d* between cells as Least Cost Path (LCP) distances. We used a transition function that yields full permeability for flat and downhill (negative) slopes over land, and decreases towards zero for uphill (positive) slope. Water was given extremely low permeability (0.001) to allow rare events of crossing water. The exact shape of the permeability function followed the equation:

$$Permeability = \begin{cases} 1, \text{if } \Delta x < 0 \\ 1/((a\Delta x)^b + 1), \text{if } \Delta x \geq 0 \end{cases} \quad \text{(eqn. 1)}$$

and was decided after visual examination of the permeability map of Europe (via comparisons to a ‘flat’ LCP distance without the effect of terrain [a = 0, b = 1], so that the Alps increased dispersal distance by approximately 500 km), as well as after visual examination of simulations of resultant LCP routes across the continent. To calculate the LCP distances, we used elevation data at a 5 × 5 km^2 resolution, and the distances were averaged within the 100 × 100 km^2 cells. We computed transitions assuming 8-cell neighbourhoods.

Supplementary References

1. Dormann, C. F. *et al.* Collinearity : a review of methods to deal with it and a simulation study evaluating their performance. *Ecography (Cop.).* **36,** 27–46 (2013).
2. New, M., Hulme, M. & Jones, P. Representing Twentieth-Century Space – Time Climate Variability. Part I: Development of a 1961 – 90 Mean Monthly Terrestrial Climatology. *J. Clim.* **12,** 829–856 (1999).
3. Michaletz, S. T., Cheng, D., Kerkhoff, A. J. & Enquist, B. J. Convergence of terrestrial plant production across global climate gradients. *Nature* **512,** 39–43 (2014).
4. Klein Goldewijk, K. & Verburg, P. H. Uncertainties in global-scale reconstructions of historical land use: An illustration using the HYDE data set. *Landsc. Ecol.* **28,** 861–877 (2013).
5. Ellis, E. C. *et al.* Used planet: a global history. *Proc. Natl. Acad. Sci. U. S. A.* **110,** 7978–85 (2013).
6. Klein Goldewijk, K., Beusen, A., Van Drecht, G. & De Vos, M. The HYDE 3.1 spatially explicit database of human-induced global land-use change over the past 12,000 years. *Glob. Ecol. Biogeogr.* **20,** 73–86 (2011).
7. Kaplan, J. O. *et al.* Holocene carbon emissions as a result of anthropogenic land cover change. *The Holocene* **21,** 775–791 (2010).
8. Kaplan, J. O., Krumhardt, K. M. & Zimmermann, N. The prehistoric and preindustrial deforestation of Europe. *Quat. Sci. Rev.* **28,** 3016–3034 (2009).
9. Sommer, R. S. & Benecke, N. The recolonization of Europe by brown bears Ursus arctos Linnaeus, 1758 after the Last Glacial Maximum. *Mamm. Rev.* **35,** 156–164 (2005).
10. Davison, J. *et al.* Late-Quaternary biogeographic scenarios for the brown bear (Ursus arctos), a wild mammal model species. *Quat. Sci. Rev.* **30,** 418–430 (2011).

11. Kopatz, A. *et al.* Admixture and gene flow from Russia in the recovering Northern European brown bear (Ursus arctos). *PLoS One* **9,** 1–10 (2014).
12. Sommer, R. S. *et al.* Late Quaternary distribution dynamics and phylogeography of the red deer (Cervus elaphus) in Europe. *Quat. Sci. Rev.* **27,** 714–733 (2008).
13. Sommer, R. S., Fahlke, J. M., Schmölcke, U., Benecke, N. & Zachos, F. E. Quaternary history of the European roe deer Capreolus capreolus. *Mamm. Rev.* **39,** 1–16 (2009).
14. Benecke, N., von den Driesch, A. & Heinrich, D. *Holozängeschichte der Tierwelt Europas*. (Datensammlung hrsg. v. IANUS, 2016). doi:10.13149/001.mcus7z-2
15. Benecke, N. The project 'The Holocene History of the European Vertebrate Fauna'. *Archäologie in Eurasien* **6,** 151–162 (1999).
16. Steyaert, S. M. J. G., Endrestøl, A., Hackländer, K., Swenson, J. E. & Zedrosser, A. The mating system of the brown bear Ursus arctos. *Mamm. Rev.* **42,** 12–34 (2012).
17. Schwartz, C. C., Miller, S. D. & Haroldson, M. A. Grizzly Bear. *Wild Mamm. North Am. Biol. Manag. Conserv.* 556–586 (2003).
18. Humphries, M. M., Thomas, D. W. & Speakman, J. R. Climate-mediated energetic constraints on the distribution of hibernating mammals. *Nature* **418,** 313–316 (2002).
19. R Development Core Team. R: A language and environment for statistical computing. R Foundation for Statistical Computing, Vienna, Austria. Available at: https://www.R-project.org/. (2016).
20. South, A. rworldxtra: Country boundaries at high resolution. R package version 1.01. https://CRAN.R-project.org/package=rworldxtra. (2012).
21. Kass, R. E. & Raftery, A. E. Bayes Factors. *J. Am. Stat. Assoc.* **90,** 773–795 (1995).
22. Zedrosser, A., Dahle, B., Støen, O. G. & Swenson, J. E. The effects of primiparity on reproductive performance in the brown bear. *Oecologia* **160,** 847–854 (2009).
23. Zedrosser, A., Steyaert, S. M. J. G., Gossow, H. & Swenson, J. E. Brown bear

conservation and the ghost of persecution past. *Biol. Conserv.* **144,** 2163–2170 (2011).

24. Frkovic, A., Huber, D. & Kusak, J. Brown bear litter sizes in Croatia. *Ursus* **12,** 103–106 (2001).

25. Wiegand, T., Stephan, T., Naves, J. & Fernandez, a. Assessing the risk of extinction for the brown bear (Ursus arctos) in the Cordillera Cantabrica, Spain. *Ecol. Monogr.* **68,** 539–570 (1998).

26. Nawaz, M. A., Swenson, J. E. & Zakaria, V. Pragmatic management increases a flagship species, the Himalayan brown bears, in Pakistan's Deosai National Park. *Biol. Conserv.* **141,** 2230–2241 (2008).

27. Mano, T. & Tsubota, T. Reproductive characteristics of brown bears on the Oshima Peninsula, Hokkaido, Japan. *J. Mammal.* **83,** 1026–1034 (2002).

28. Kohira, M., Okada, H., Nakanishi, M. & Yamanaka, M. Modeling the effects of human-caused mortality on the brown bear population on the Shiretoko Peninsula, Hokkaido, Japan. *Ursus* **20,** 12–21 (2009).

29. Kansas, J. L. Status of the grizzly bear (Ursus arctos) in Alberta. *Wild* (2002).

30. Ferguson, S. H. & Mcloughlin, P. D. Effect of energy availability, seasonality, and geographic range on brown bear life history. *Ecography (Cop.).* **23,** 193–200 (2000).

31. Ciarniello, L. M., Boyce, M. S., Seip, D. R. & Heard, D. C. Demographics in Wilderness Mountains Versus a Plateau with Resource Development. *Wildlife Biol.* **15,** 247–265 (2009).

32. Case, R. L. & Buckland, L. Reproductive Characteristics Of Grizzly Bears In The Kugluktuk Area, Northwest Territories, Canada. *Ursus* **10,** 41–47 (1995).

33. Canfield, J. & Harting, A. in *Grizzly Bear Compendium* (eds. LeFranc Jr., M. N., Moss, M. B., Patnode, K. A. & Sugg, W. C. I.) 47–56 (Interagency Grizzly Bear Committee, 1987).

34. Garshelis, D. L., Gibeau, M. & Herrero, S. Grizzly bear demographics in and around Banff National Park and Kananaskis country, Alberta. *J. Wildl. Manage.* **69,** 277–297 (2005).

35. Nagy, J. A. S. & Haroldson, M. A. Comparisons of some home range and population parameters among four grizzly bear populations in Canada. *Int. Conf. Bear Res. Manag.* **8,** 227–235 (1990).

36. Wakkinen, W. L. & Kasworm, W. F. Demographics and population trends of grizzly bears in the Cabinet–Yaak and Selkirk Ecosystems of British Columbia, Idaho, Montana, and Washington. *Ursus* **15,** 65–75 (2004).

37. Kasworm, W. F. *et al. Cabinet-Yaak Grizzly Bear Recovery Area 2014 Research and Monitoring Progress Report*. (2015).

38. Ballard, W., Ayers, L., Reed, D., Fancy, S. & Roney, K. *Demography of grizzly bears in relation to hunting and mining development in northwestern Alaska*. (1993).

39. Sellers, R. A. & Aumiller, L. D. Brown bear population characteristics at McNeil River, Alaska. *Int. Conf. Bear Res. Manag.* **9,** 283–293 (1994).

40. Miller, S. D., Sellers, R. A. & Keay, J. A. Effects of hunting on brown bear cub survival and litter size in Alaska. *Ursus* **14,** 130–152 (2003).

41. Van Daele, L. J. *et al.* Grizzlies, Eskimos, and biologists: cross-cultural bear management in southwest Alaska. *Ursus* **12,** 141–152 (2001).

42. Mace, R. D. & Waller, J. S. Demography and population trend of Grizzly bears in the Swan Mountains, Montana. *Conserv. Biol.* **12,** 1005–1016 (1998).

43. Mace, R. D. & Waller, J. S. *Final Report: Grizzly bear ecology in the Swan Mountains*. (1997).

44. Kovach, S. D. *et al.* Reproduction and Survival of Brown Bears in Southwest Alaska, USA. *Ursus* **17,** 16–29 (2016).

Supplementary Figures

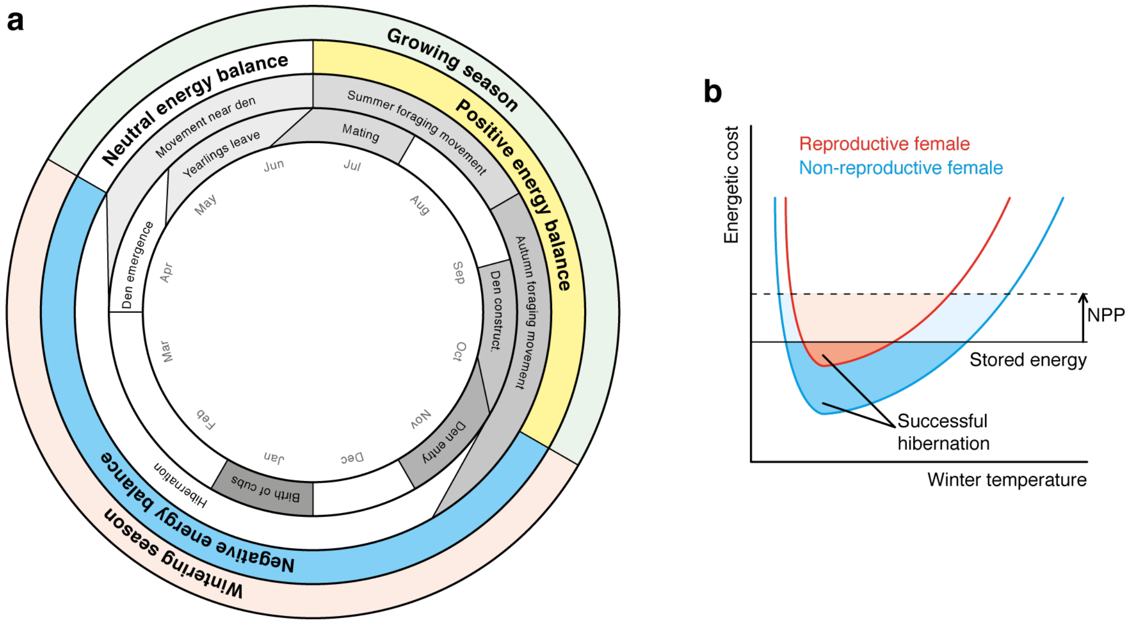

Supplementary Figure 1. Schematic representation of the annual cycle of the brown bear and the resulting life-history trade off. a, The annual cycle of the brown bear representative for a population in central Sweden. Bears emerge from the den in spring. Yearlings separate from females before the mating season. After fertilization embryos develop to the blastocyst stage, but development is delayed until implantation when females enter the den[16]. The effective gestation period is six to eight weeks and females typically give birth to one to three cubs in their den. Brown bears pass through three biochemical and physiological stages in their active period, changing from low food intake (*hypophagia*) in spring, to normal food intake in summer, to high food intake (*hyperphagia*) in autumn. The intake of high-energy foods such as seeds, nuts and berries during late summer and autumn is particularly important[17], as this is the period of accumulation of adipose tissue (*positive energy balance*) that is essential for hibernation and reproduction during the wintering season (*negative energy balance*). **b**, The hypothetical relationship between winter temperature and total winter energy requirements predicted by bioenergetic models[18], shown separately for reproductive (red) and non-reproductive (blue) female brown bears. The horizontal lines indicate the amount of energy stored at the onset of hibernation in a year with low (solid line) and high (dashed line) net primary productivity (NPP) during the growing season. Successful hibernation (i.e., survival) is only possible if energetic costs of hibernation (and reproduction) do not exceed stored energy. The range of winter temperatures allowing successful hibernation is much narrower in reproductive (red shaded area) compared with non-reproductive (blue shaded area) females and in years with low compared with high net primary productivity.

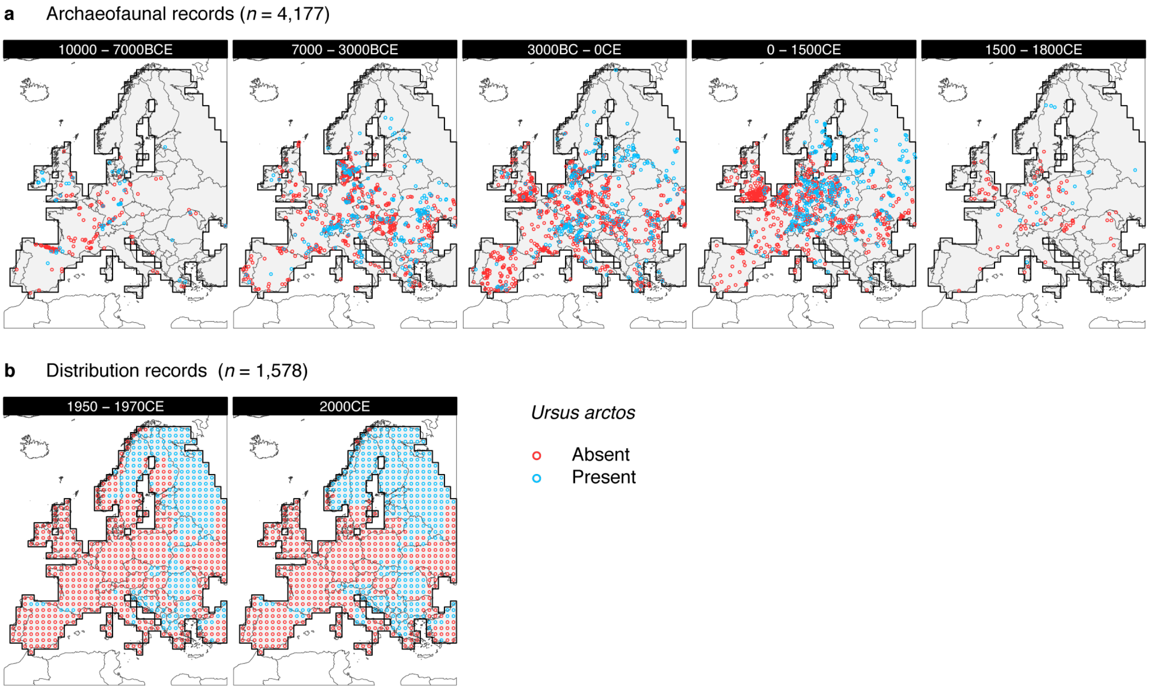

Supplementary Figure 2. Detection of the brown bear in Europe during the past 12,000 years. a,**b**, Maps showing **a**, the distribution of the archaeofaunal records (*n* = 4,177), and **b**, contemporary occurrence records (*n* = 1,578). Circles represent individual archaeofaunal and contemporary occurrence records with (blue) and without (red) detection of brown bears. The maps in (**a**, **b**) were created with the statistical programming language *R*[19] using the package *rworldxtra*[20].

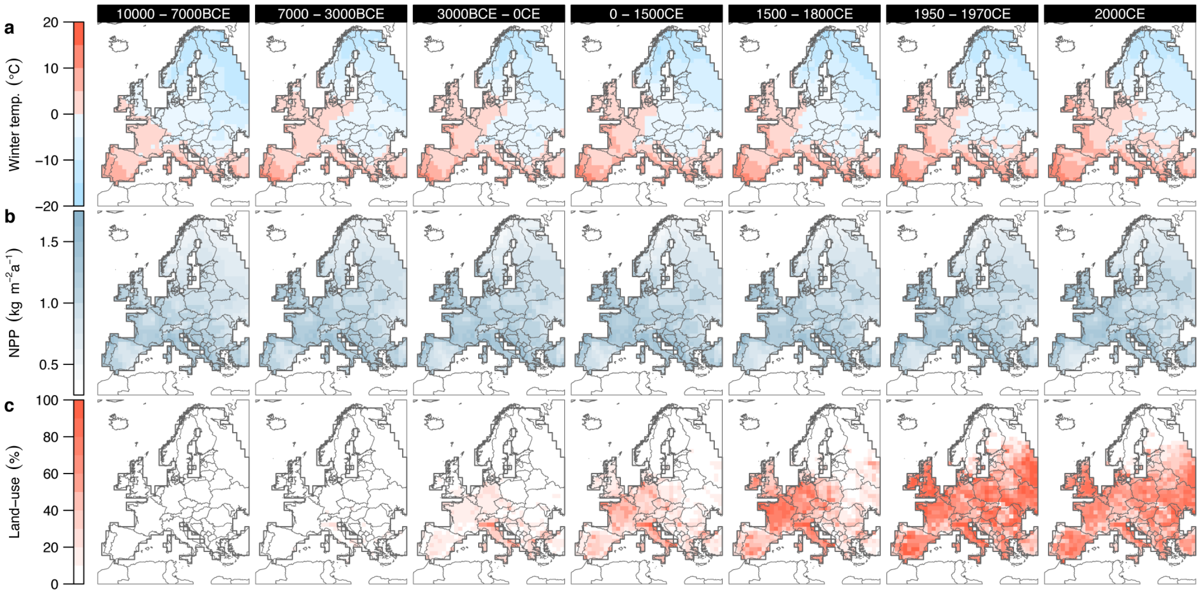

Supplementary Figure 3. Spatiotemporal changes in winter temperature, net primary productivity and land use in Europe during the past 12,000 years. a–**c**, Maps showing changes in **a**, winter temperature (°C), **b**, net primary productivity (kg m^{-2} a^{-1}) and **c**, land use (%). In **c**, land use averaged across the two land-use scenarios (see Supplementary Methods and Supplementary Fig. 4) is shown. The maps in (**a**, **b**) were created with the statistical programming language *R*[19] using the package *rworldxtra*[20].

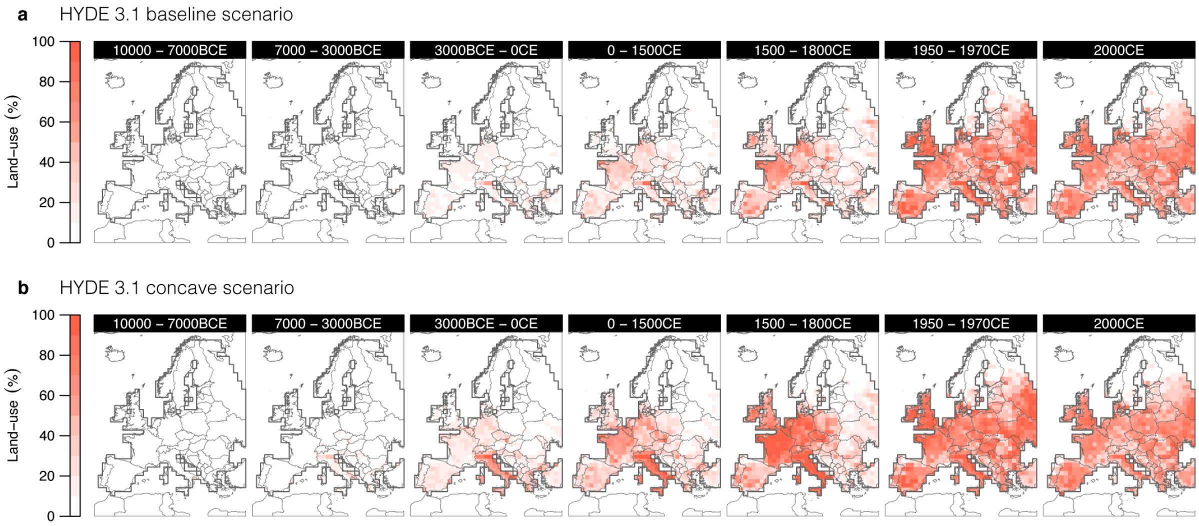

Supplementary Figure 4. Reconstructed spatiotemporal changes in land use in Europe during the past 12,000 years according to two scenarios of the HYDE 3.1 model[4–6]. a, **b**, Maps showing changes in land use (%) according to **a**, the baseline scenario and according to **b**, the concave scenario of the HYDE 3.1 model. The maps in (**a**, **b**) were created with the statistical programming language *R*[19] using the package *rworldxtra*[20].

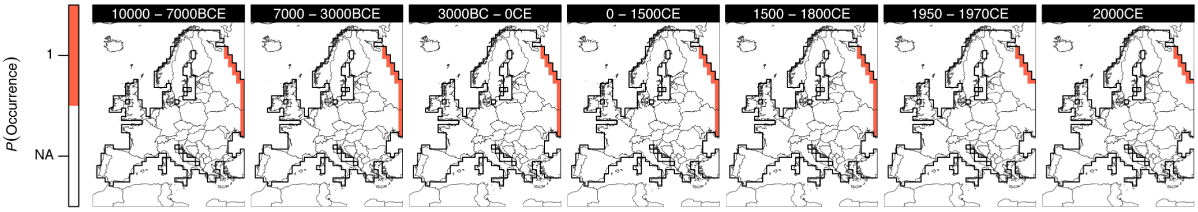

Supplementary Figure 5. Implementation of a permanent source population at the eastern border of the study area. Map showing the cells of the metapopulation model that were fixed to an occupancy state of 1 (i.e., occupied) to allow for constant colonization of the European continent by the brown bear from Asia (Scenario: colonization yes). In the scenario without this assumption the cells were not informed *a priori* so that the model estimated the occupancy states of these cells. The maps in (**a**, **b**) were created with the statistical programming language *R*[19] using the package *rworldxtra*[20].

Supplementary Tables

Supplementary Table 1. Summary of generalized linear mixed effects models testing for the effects of female body mass, three variables describing the growing season and mean temperature during the winter season (T_{ws}) on the reproductive rate of female brown bears (n = 38 populations).

Source of variance	estimate	s.e.m.	t	p	$R^2_{marginal}$	vif
(**a**) Fixed effects						
Intercept	0.78	0.23	3.35	0.00081		
log(female body mass)	0.054	0.019	2.94	0.0033	0.13	1.41
Net primary productivity	0.21	0.044	4.72	< 0.0001	0.35	7.32
T_{ws}	-0.19	0.047	-3.99	< 0.0001	0.25	8.16
Random effect (SD)						
Continent	0.28					
Residuals	0.50					
(**b**) Fixed effects						
Intercept	0.81	0.22	3.68	0.00023		
log(female body mass)	0.087	0.019	4.67	< 0.0001	0.25	1.73
Growing degree days	0.14	0.023	6.09	< 0.0001	0.50	2.02
T_{ws}	-0.10	0.026	-3.94	< 0.0001	0.17	2.93
Random effect (SD)						
Continent	0.26					
Residuals	0.47					
(**c**) Fixed effects						
Intercept	0.85	0.22	3.95	< 0.0001		
log(female body mass)	0.11	0.020	5.52	< 0.0001	0.32	2.19
T_{gs}	0.12	0.018	6.94	< 0.0001	0.56	1.89
T_{ws}	-0.10	0.024	-4.12	< 0.0001	0.16	3.03
Random effect (SD)						
Continent	0.24					
Residuals	0.44					

To assess the robustness of the relationships, we performed this analysis with three variables describing the growing season (**a**, net primary productivity; **b**, growing degree days; and **c**, temperature during the growing season, T_{gs}). The model was fitted using a *gamma*-distribution with an *identity*-link function for reproductive rate. Given are standardized parameter estimates with their standard error (s.e.m.). For the random effect and the residuals the standard deviations (SD) are given. The $R^2_{marginal}$ was obtained by comparing the fit of the full model with the fit of a reduced model without the term in question. We assessed whether the analysis was affected by collinearity among predictors, by calculating variance inflation factors (VIFs) and by using alternative variables to characterize the growing season (see Supplementary Methods). Generally VIF-values above a threshold of 10 indicate that collinearity might adversely affect regression results[1]. Using alternative variables to characterize the growing season in the analysis reduced VIFs from 8 to 3 but led to identical conclusions, suggesting that collinearity does not affect the conclusions from this analysis.

Supplementary Table 2. Summary of the path analysis with Bayesian model selection.

	Path X, Y (from X to Y)										
Model No.	ELE, WT	**WT, NPP**	ELE, NPP	NPP, LU	**WT, LU**	ELE, LU	**LU, EXT**	**NPP, EXT**	**WT, EXT**	**ELE, EXT**	*P*(Sel)
1012	x	**x**			**x**	x	**x**	**x**	**x**	**x**	0.32
988	x	**x**		x	**x**		**x**	**x**	**x**	**x**	0.24
1020	x	**x**		x	**x**	x	**x**	**x**	**x**	**x**	0.092
1016	x	**x**	x		**x**	x	**x**	**x**	**x**	**x**	0.086
992	x	**x**	x	x	**x**		**x**	**x**	**x**	**x**	0.079
980	x	**x**			**x**		**x**	**x**	**x**	**x**	0.050
1011		**x**			**x**	x	**x**	**x**	**x**	**x**	0.038
1024	x	**x**	x	x	**x**	x	**x**	**x**	**x**	**x**	0.031
987		**x**		x	**x**		**x**	**x**	**x**	**x**	0.026
984	x	**x**	x		**x**		**x**	**x**	**x**	**x**	0.015
1015		**x**	x		**x**	x	**x**	**x**	**x**	**x**	0.010
1019		**x**		x	**x**	x	**x**	**x**	**x**	**x**	0.0055
979		**x**			**x**		**x**	**x**	**x**	**x**	0.0048
991		**x**	x	x	**x**		**x**	**x**	**x**	**x**	0.0028
1023		**x**	x	x	**x**	x	**x**	**x**	**x**	**x**	0.0025
983		**x**	x		**x**		**x**	**x**	**x**	**x**	0.0020
P(Sel)	0.91	**1.00**	0.23	0.47	**1.00**	0.59	**1.00**	**1.00**	**1.00**	**1.00**	
$2\log_e$(BF)	4.63	**>10***	-2.46	-0.22	**>10***	0.70	**>10***	**>10***	**>10***	**>10***	
Post. mean	0.12	**0.71**	-0.028	0.047	**0.39**	-0.053	**0.79**	**-0.48**	**1.3**	**-0.31**	
2.5% CI	0.012	**0.68**	-0.087	-0.010	**0.34**	-0.10	**0.60**	**-0.72**	**1.1**	**-0.42**	
97.5% CI	0.19	**0.73**	0.006	0.12	**0.46**	-0.008	**0.99**	**-0.25**	**1.5**	**-0.20**	

The model tested for the direct and indirect effects of elevation (ELE), winter temperature (WT), net primary productivity (NPP) and land use (LU) on the extinction rate (EXT) of the European brown bear during the Holocene. List of the path models selected during the Markov Chain Monte Carlo search, along with the marginal selection probabilities of the path models [*P*(Sel)], and posterior means and 95 % credible intervals of the hypothesized paths, as well as the selection probabilities and Bayes Factors[21] [$2\log_e$(BF)] as a measure of support for each path. Paths with decisive support ($2\log_e$(BF) > 10) are highlighted in boldface type. Note that positive effects increase extinction rate, whereas negative effects decrease extinction rate. Uncertainty in model assumptions was incorporated by a two-factorial design with two scenarios for changes in per capita land-use intensity during the Holocene (constant *versus* decreasing) and two scenarios for colonization of the European continent by the brown bear from Asia (yes *versus* no), respectively (see Supplementary Methods and Supplementary Figs. 4 and 5).

Supplementary Table 3. Data on life histories of female brown bears from 43 populations.

continent	country	locality	lon	lat	LI	LS	RR	FM	nLI	nLS	nFM	T_{gs}	T_{ws}	NPP	GDD	MAT	MAP	Ref LS	Ref LI	Ref FM
Europe	Sweden	Noppikoski and Älvdalen, Kopparberg county	18.0	61.0	1.74	2.29	1.32	117	126	136	59	14.100	-3.860	0.910	1169	5.1	545.0	22	22	23
Europe	Sweden	Kvikkjokk, Norrbotten county	18.0	67.0	2.44	2.43	1.00	120	59	75	46	10.940	-11.110	0.540	446	-1.7	528.7	22	22	23
Europe	Croatia	Dinara Mountains	16.4	44.1	2.06	2.39	1.16	128	17	56	67	15.940	-0.910	1.380	1975	9.7	963.6	24	24	23
Europe	Spain	Western Cantabrian Mountains	-5.0	43.0	3.30	2.26	0.68	94	NA	23	12	15.280	3.600	1.130	2050	10.7	714.0	25	16	23
Europe	Finland and Russia	Finish/Russian Karelia	33.6	63.7	NA	2.50	NA	132	NA	31	81	13.660	-8.610	0.710	997	1.2	572.2	23	-	23
Europe	Russia	Leningrad Oblast	31.3	59.9	NA	2.35	NA	127	NA	31	15	13.840	-7.500	0.920	1386	4.2	620.3	23	-	23
Asia	Pakistan	Deosai National Park	75.5	35.0	5.70	1.33	0.23	73	24	33	4	13.040	-9.540	0.700	935	1.6	400.1	26	26	26
Asia	Japan	Oshima Peninsula, Southern Hokkaido	140.3	41.9	2.30	1.62	0.70	102	30	13	17	16.610	-3.090	1.310	1945	8.9	1330.9	27	27	23
Asia	Japan	Teshio District, Northern Hokkaido	141.9	45.0	NA	1.59	NA	103	NA	32	31	14.240	-5.300	1.080	1545	6.2	1228.2	28	-	23
Asia	Russia	Sakha (Yakutia)	129.2	66.4	NA	1.89	NA	142	NA	119	NA	10.620	-34.220	0.090	232	-18.4	371.0	23	-	23
North Amerika	Canada	Rocky Mountain Foothills, Westcentral Alberta	-123.0	56.5	4.00	1.90	0.48	146	1	5	8	12.100	-12.540	0.560	696	-1.4	528.9	29	30	30
North Amerika	Canada	Parsnip River, British Columbia	-122.6	54.7	3.50	1.95	0.56	113	2	20	29	13.150	-9.250	0.710	808	1.2	654.3	31	31	31
North Amerika	Canada	Glacier National Park, British Columbia	-113.7	48.7	NA	1.70	NA	NA	NA	35	NA	15.180	-7.340	0.790	1224	4.2	461.3	17	-	-
North Amerika	Canada	Richardson River, Kugluktuk	-115.5	67.9	3.30	2.26	0.68	126	6	19	60	9.720	-19.640	0.200	246	-11.0	249.0	32	30	30
North Amerika	Canada	Anderson Horton Rivers, Inuvialuit Region	-133.7	68.4	4.90	2.27	0.46	105	24	NA	NA	10.550	-21.540	0.220	433	-10.2	190.3	32	30	30
North Amerika	Canada	Tuktoyaktuk Peninsula/Richards Island	-133.0	69.4	3.30	2.30	0.70	124	8	28	36	10.550	-21.540	0.220	433	-10.2	190.3	33	30	30
North Amerika	Canada	Vuntut National Park, Northern Yukon	-140.0	68.5	3.50	2.00	0.57	116	4	6	35	7.450	-20.930	0.170	75	-12.7	165.9	33	33	30
North Amerika	Canada	Kluane National Park, Yukon	-138.3	60.6	3.10	1.70	0.55	121	NA	11	35	8.520	-13.910	0.340	179	-6.2	576.8	17	30	30
North Amerika	Canada	Mackenzie Mountains, Northwest Territories	-128.0	64.0	3.80	1.83	0.48	110	11	6	28	9.930	-18.580	0.260	294	-8.7	492.8	33	33	30
North Amerika	Canada	Banff National Park and Kananaskis Country	-115.0	51.0	4.40	1.84	0.42	120	15	38	17	12.160	-8.550	0.660	608	0.4	578.9	34	34	30
North Amerika	Canada	Jasper National Park	-118.1	52.8	3.50	2.00	0.57	129	NA	3	7	10.700	-8.650	0.620	440	-0.3	713.4	33	35	30
North Amerika	Canada	Selkirk Mountains, British Columbia	-117.0	49.0	3.00	2.18	0.73	123	8	17	21	14.190	-6.200	0.910	989	4.1	758.1	36	36	37
North Amerika	USA	Cabinet-Yaak, Northwest Montana	-116.0	48.0	3.00	2.07	0.69	123	7	14	21	15.770	-5.420	0.950	1425	4.6	651.0	36	36	37
North Amerika	USA	Flathead, Montana	-114.0	48.3	3.10	2.20	0.71	114	17	26	16	14.300	-7.690	0.830	977	3.0	692.5	17	17	17
North Amerika	USA	Western Brooks Range, Alaska	-160.8	68.9	4.10	2.03	0.50	117	16	23	35	7.610	-17.530	0.220	91	-10.2	189.7	33	30	30
North Amerika	USA	Eastern Brooks Range, Alaska	-149.5	68.2	4.24	1.78	0.42	108	NA	13	31	9.950	-22.740	0.190	312	-11.5	205.0	33	33	30
North Amerika	USA	Noatak River, Northwest Alaska	-162.5	67.0	3.30	2.17	0.66	132	10	35	NA	10.280	-15.410	0.330	358	-6.5	244.5	38	38	30
North Amerika	USA	McNeil River State Game Sanctuary	-154.3	59.1	3.94	2.15	0.55	160	35	137	NA	11.380	-7.030	0.670	552	0.6	780.5	39	39	30
North Amerika	USA	Katmai National Park, Shelikof Strait coast	-156.4	58.7	5.60	2.06	0.37	213	19	51	59	11.620	-8.200	0.670	690	0.6	550.2	40	30	30
North Amerika	USA	Kodiak Island, Alaska	-153.4	57.5	4.60	2.50	0.54	202	41	29	16	10.090	-2.430	0.870	621	3.5	1412.2	17	17	17
North Amerika	USA	Admiralty Island	-134.3	57.7	3.90	1.80	0.46	169	7	32	18	10.700	-1.980	0.970	678	4.8	2465.9	17	17	17
North Amerika	USA	Nelchina Basin	-146.8	62.0	3.80	2.10	0.55	144	44	64	21	10.260	-9.740	0.560	551	-1.4	946.3	17	17	17
North Amerika	USA	Black Lake, Alaska	-159.0	56.0	3.00	2.57	0.86	256	NA	46	34	9.680	-3.350	0.850	528	3.2	673.7	40	33	23
North Amerika	USA	Alaska Range, Alaska	-151.0	63.1	4.00	2.20	0.55	154	51	36	52	11.950	-13.590	0.430	468	-3.9	529.1	17	30	30
North Amerika	USA	Kuskokwim Mountains, Southwestern Alaska	-159.0	60.0	4.53	1.94	0.43	170	34	33	23	11.060	-9.070	0.620	608	-0.2	601.7	41	41	30
North Amerika	USA	Swan Mountains	-113.6	47.7	3.00	1.64	0.55	112.64	6	17	6	15.060	-6.900	0.820	1299	3.4	483.3	42	42	43
North Amerika	USA	East Front, Montana	-110.0	47.0	2.60	2.20	0.85	125	11	41	6	18.400	-6.940	0.680	1793	6.4	386.3	17	17	17,23
North Amerika	USA	Yellowstone National Park 1959-1970	-110.5	44.6	3.20	2.20	0.69	152	68	173	72	14.330	-8.640	0.750	1181	1.8	575.3	17	17	17
North Amerika	USA	Yellowstone National Park 1975-1989	-110.5	44.6	2.60	1.90	0.73	134	20	232	63	14.330	-8.640	0.750	1181	1.8	575.3	17	17	17
North Amerika	USA	Mission Mountains	-113.9	47.4	3.30	2.12	0.64	127	NA	NA	3	15.060	-6.900	0.820	1299	3.4	483.3	33	30	30
North Amerika	USA	Alaska Peninsula, Alaska	-158.8	56.5	3.00	2.30	0.77	226	81	200	63	9.860	-4.440	0.760	583	2.0	572.4	17	17	17
North Amerika	USA	Denali National Park, Alaska	-151.2	63.3	2.10	2.10	1.00	125	NA	42	65	11.950	-13.590	0.430	468	-3.9	529.1	40	44	23
North Amerika	USA	Susitna River, Talkeetna, Southcentral Alaska	-150.1	62.3	2.10	2.09	1.00	170	NA	91	50	11.950	-13.590	0.430	468	-3.9	529.1	40	44	23

lon and lat, longitude and latitude (decimal degree); LI, inter-birth interval (years); LS, litter size (cubs female^{-1}); RR, reproductive rate (cubs female^{-1} year^{-1}); FM, female mass (kg); nLI, nLS, nFM, sample sizes for the three life-history variables; T_{gs}, temperature of the growing season; T_{ws}, temperature of the winter season; NPP, net primary productivity (kg m^{-2}a^{-1}); GDD, growing degree days (base 5 °C); MAT, mean annual temperature (°C); MAP, total annual precipitation (mm); Ref LS, Ref LI, Ref FM, codes of the references for the three life-history variables.

Supplementary Computer Code

Supplementary Computer Code 1. JAGS code of the metapopulation model.

Black text indicates the model code that is compiled by JAGS, # comments are highlighted in blue.

```
var wM[ncell, ncell], swM[ncell, ncell], cM[ncell, ncell, ntime];
model {
    ##### priors for SSVS variable selection
    sd.beta ~ dunif(0, 100)
    tau.beta <- pow(sd.beta, -2)
    for (i in 1:10) {
        # prior for inclusion probability of variables in path model
        g[i] ~ dbern(0.5)
        # indicator variable selection for path model
        tau.b[i] <- (1 - g[i]) * 3600 + g[i] * tau.beta
    }
    # indicator for ID of selected model
    mod.id <- 1 + g[1] * 1 + g[2] * 2 + g[3] * 4 + g[4] * 8 + g[5] * 16 + g[6] * 32 + g[7] *
64 + g[8] * 128 + g[9] * 256 + g[10] * 512
    # priors for cell specific random effects
    for (s in 1:ncell) {
        s.clw[s] ~ dnorm(int.clw, tau.s.clw)
        s.npp[s] ~ dnorm(int.npp, tau.s.npp)
        s.lu[s] ~ dnorm(int.lu, tau.s.lu)
    }
    ##### priors for winter temperature regression
    int.clw ~ dnorm(0, 0.001)
    beta.alt.clw ~ dnorm(0, tau.b[1])
    sd.clw ~ dunif(0, 100)
    tau.clw <- pow(sd.clw, -2)
    # cell-specific random effect
    sd.s.clw ~ dunif(0, 100)
    tau.s.clw <- pow(sd.s.clw, -2)
    ##### priors for net primary productivity regression
    int.npp ~ dnorm(0, 0.001)
    beta.clw.npp ~ dnorm(0, tau.b[2])
    beta.alt.npp ~ dnorm(0, tau.b[3])
    sd.npp ~ dunif(0, 100)
    tau.npp <- pow(sd.npp, -2)
    # cell-specific random effect
    sd.s.npp ~ dunif(0, 100)
    tau.s.npp <- pow(sd.s.npp, -2)
    ##### priors for land use regression
    int.lu ~ dnorm(0, 0.001)
    beta.npp.lu ~ dnorm(0, tau.b[4])
    beta.clw.lu ~ dnorm(0, tau.b[5])
    beta.alt.lu ~ dnorm(0, tau.b[6])
    sd.lu ~ dunif(0, 100)
    tau.lu <- pow(sd.lu, -2)
    # cell-specific random effect
    sd.s.lu ~ dunif(0, 100)
    tau.s.lu <- pow(sd.s.lu, -2)
    ##### priors for metapopulation model
    # prior for initial occupancy
    muz1 ~ dbeta(10, 1)
    # priors for extinction rate regression
    int.phi ~ dnorm(0, 0.001)
    beta.lu.phi ~ dnorm(0, tau.b[7])
    beta.npp.phi ~ dnorm(0, tau.b[8])
    beta.clw.phi ~ dnorm(0, tau.b[9])
    beta.alt.phi ~ dnorm(0, tau.b[10])
    # prior for shape parameter of dispersal kernel
    alpha ~ dgamma(3, 1)
    ##### priors for detection model
    beta.nsp.pdet ~ dnorm(0, 0.001)
    for (i in 1:ntype) {
```

```
        # means parameterization for site types
        beta.typ.pdet[i] ~ dnorm(0, 0.001)
    }
    ##### distance-dependent influence of cells on connectivity
    # weights matrix
    wM[ncell, ncell] <- 0
    for (s1 in 1:(ncell - 1)) {
        wM[s1, s1] <- 0
        for (s2 in (s1 + 1):ncell) {
            wM[s1, s2] <- exp(-alpha * dM[s1, s2])
            wM[s2, s1] <- wM[s1, s2]
        }
    }
    for (s in 1:ncell) {
        # standardized weights matrix
        swM[s, ] <- wM[s, ]/sum(wM[s, ])
        # stochastic node for occurrence at t = 1
        z[s, 1] ~ dbern(muz1)
        # connectivity matrix at t = 1
        cM[s, , 1] <- z[s, 1] * swM[s, ]
    }
    for (t in 1:(ntime - 1)) {
        for (s in 1:ncell) {
            # winter temperature regression
            muclw[s, t] <- s.clw[s] + beta.alt.clw * alt[s, t]
            clw[s, t] ~ dnorm(muclw[s, t], tau.clw)
            # net primary productivity regression
            munpp[s, t] <- s.npp[s] + beta.clw.npp * clw[s, t] + beta.alt.npp * alt[s, t]
            npp[s, t] ~ dnorm(munpp[s, t], tau.npp)
            # land use regression
            mulu[s, t] <- s.lu[s] + beta.npp.lu * npp[s, t] + beta.clw.lu * clw[s, t] +
beta.alt.lu * alt[s, t]
            lu[s, t] ~ dnorm(mulu[s, t], tau.lu)
            # extinction rate
            log(r.phi[s, t]) <- int.phi + beta.lu.phi * lu[s, t] + beta.npp.phi * npp[s, t] +
beta.clw.phi * clw[s, t] + beta.alt.phi * alt[s, t]
            # colonization rate
            log(r.gam[s, t]) <- sum(cM[, s, t])
            # Kolmogorov forward equations map rates to probabilities (equivalent to matrix
exponentiation)
            p.phi[s, t] <- 1 - ((r.gam[s, t] + r.phi[s, t] * exp(-(r.phi[s, t] + r.gam[s, t])
* time[t]))/(r.phi[s, t] + r.gam[s, t]))
            p.gam[s, t] <- 1 - ((r.phi[s, t] + r.gam[s, t] * exp(-(r.phi[s, t] + r.gam[s, t])
* time[t]))/(r.phi[s, t] + r.gam[s, t]))
            # metapopulation model
            muz[s, t + 1] <- z[s, t] * (1 - p.phi[s, t]) + (1 - z[s, t]) * p.gam[s, t]
            # stochastic node for occurrence at t = 2...7
            z[s, t + 1] ~ dbern(muz[s, t + 1])
            # connectivity matrix at t = 2...7
            cM[s, , t + 1] <- z[s, t + 1] * swM[s, ]
        }
    }
    for (i in 1:nsamp1) {
        # imputation of excavation site types based on relative frequency in each time period
        site.type[i] ~ dcat(stpM[1:ntype, x1[i, 2]])
        # observation model
        logit(pdet[i]) <- beta.typ.pdet[site.type[i]] + beta.nsp.pdet * nsp[i]
        # multiplication of 'true' occurrence in a given cell with detection probability
        muy[i] <- z[x1[i, 1], x1[i, 2]] * pdet[i]
        # link to archeofaunal records from excavation sites
        y1[i] ~ dbern(muy[i])
    }
    for (i in 1:nsamp2) {
        # link to recent distribution records (assuming perfect detection)
        y2[i] ~ dbern(z[x2[i, 1], x2[i, 2]])
    }
}
```
